# Simplified, minimally invasive, beating-heart technique for redo isolated tricuspid valve surgery

**DOI:** 10.1186/s13019-020-01192-1

**Published:** 2020-06-18

**Authors:** Shuyang Lu, Kai Song, Wangchao Yao, Limin Xia, Lili Dong, Yongxin Sun, Tao Hong, Shouguo Yang, Chunsheng Wang

**Affiliations:** 1 Department of Cardiovascular Surgery, Zhongshan Hospital, Fudan University, The Shanghai Institute of Cardiovascular Diseases, No. 1609 Xietu Road, Xuhui District, Shanghai, 200032 China; 2 Department of Echocardiography, Zhongshan Hospital, Fudan University, Shanghai, 200032 China

**Keywords:** Tricuspid valve surgery, Minimally invasive surgery, Redo

## Abstract

**Background:**

Redo isolated tricuspid valve surgery is associated with a high morbidity and mortality, and its optimal timing remains controversial. Hence, here we reviewed the early and midterm results of simplified, minimally invasive, beating-heart technique for redo isolated tricuspid valve surgery in patients at high risk.

**Methods:**

A total of 32 consecutive patients underwent a redo isolated tricuspid valve surgery using minimally invasive, beating-heart technique through a right lateral thoracotomy in our center between June 2016 and April 2020. The mean age of patients was 57.4 ± 8.3 years, and 18 patients (56.3%) were women. The mean preoperative EuroSCORE was 7.8 ± 1.4 (range: 6–11). Follow-up was 87.1% complete, with a mean duration of 26.3 ± 12.3 months.

**Results:**

Both in-hospital and 30-day mortalities were 3.1%. Tricuspid valve replacement with bioprosthesis was performed in 30 patients (93.8%), and the remaining two patients (6.2%) underwent tricuspid repair (annuloplasty and leaflet reconstruction). The mean cardiopulmonary bypass time was 81.5 ± 29.0 min. The overall in-hospital duration and intensive care unit (ICU) times were 13.6 ± 7.6 days and 4.1 ± 2.8 days, respectively. Postoperative complications included prolonged ventilation in six patients (18.8%), acute kidney injury in three patients (9.4%), and neurologic event, wound infection, or permanent third-degree atrioventricular block, in one patient (3.1%) each. A total of 96.9% patients were discharged uneventfully. Four patients were lost to follow-up; there were no midterm deaths in patients who were followed up.

**Conclusions:**

Simplified, minimally invasive, beating-heart technique for redo tricuspid valve surgery is both feasible and safe, and the early and midterm results are excellent.

## Introduction

Tricuspid valve disease has always been considered less clinically important than mitral and aortic valve pathology, and its optimal timing for surgical intervention remains controversial [1]. Many patients undergo concomitant tricuspid valve surgery at the time of mitral or aortic valve intervention, while isolated tricuspid valve surgery is rarely performed [2]. However, in a large number of cases, secondary isolated tricuspid valve regurgitation develops after previous cardiac operations, which is neglected and undertreated. Considering low pressure and low resistance of the right-sided cardiac valves, patients with tricuspid valve regurgitation usually do not have significant symptoms at an early stage; therefore, they are rarely referred for isolated surgical intervention of the tricuspid valve until right heart failure, uncontrolled ascites, or edema of lower extremities develop [3, 4]. In this context, isolated tricuspid valve surgery is often associated with a high morbidity and mortality [5–7].

Median sternotomy is the standard approach for redo surgery and in patients where the tricuspid valve must be treated. However, as the development of minimally invasive surgery for atrioventricular valves, atrial septal defects, and atrial fibrillation, this technique has also been used in redo surgeries [8, 9]. Some centers have reported their experience with minimally invasive tricuspid valve surgery [10–12], but the details of surgical techniques (such as arterial inflow, venous drainage, aortic cross-clamping or not, and cooling temperature) varied among different centers. One of the questions requiring attention is whether redo tricuspid valve surgery can be successfully performed in a simplified way. During the past several years, our center modified the traditional surgical techniques and applied them in a group of patients at high risk. The early and midterm results were encouraging and both in-hospital and 30-day mortalities were dramatically reduced.

The primary objective of this study was to evaluate the safety and feasibility of our simplified redo isolated tricuspid valve surgery on the beating heart through a minimally invasive approach. The secondary objective was to summarize our early and midterm results of management of high-risk isolated tricuspid valve regurgitation.

## Patients and methods

The study protocol was approved by the Ethics Committee for the Protection of Human Subjects at the Zhongshan Hospital, Fudan University, Shanghai, China. Individual patient informed consent was not required in this study. The study was designed as a retrospective, observational study.

### Patient demographic and clinical characteristics

A total of 32 consecutive patients who had undergone redo isolated tricuspid valve operations with minimally invasive, beating-heart technique through a right lateral thoracotomy at Zhongshan Hospital, Fudan University, between June 2016 and April 2020, were reviewed retrospectively. The mean age of patients was 57.3 ± 8.7 years (range, 43–72 years), and 18 patients (56.3%) were women.

The indications for tricuspid valve operation in our study were the presence of severe or more tricuspid regurgitation along with New York Heart Association (NYHA) class III–IV, or other signs of right-sided heart failure, including uncontrolled pedal edema, ascites, pleural effusion, biological valve failure, and mechanical tricuspid valve dysfunction. We did not include patients who had undergone median open thoracotomy for the first tricuspid valve operation (*n* = 6), median redo tricuspid valve surgery (*n* = 1), and minimally invasive first tricuspid valve surgery (*n* = 1).

All patients had medical history of previous cardiac operations, including first-time or second-time reoperative cardiac procedures. All previous cardiac operations were performed through a median sternotomy. Fifteen patients (46.9%) had previously undergone a mitral valve replacement, followed by five patients (15.6%) with mitral valve replacement and tricuspid valve repair, and nine patients (28.1%) with replacement of mitral and aortic valves. Detailed surgical procedures performed in previous operations are summarized in Table 1.

**Table 1:** Surgical procedures performed in previous operations

Surgical procedures	All patients (***n*** = 32)
MVR	15 (46.9%)
TVR	1 (3.1%)
MVR + TVRep	5 (15.6%)
MVR + TVR	1 (3.1%)
MVR + AVR	9 (28.1%)
CABG	1 (3.1%)

*MVR* Mitral valve replacement, *TVR* tricuspid valve replacement, *TVRep* tricuspid valve repair, *AVR* aortic valve replacement, *CABG* coronary artery bypass grafting

Pathological characteristics of the tricuspid valve included annulus dilatation, leaflet restriction, and thickening associated with severe or more tricuspid regurgitation in the majority patients (30 patients, 93.8%), whereas two patients displayed mechanical and biological valve failure after tricuspid valve replacement.

### Surgical technique

Although techniques of minimally invasive tricuspid valve surgery have been already reported elsewhere, there are still many differences in the way it is conducted in our center. Following induction of anesthesia and intubation with a dual-lumen endotracheal tube, all patients were routinely placed with transesophageal echocardiography (TEE) and implanted with an endocardial temporary pacing lead through the right internal jugular vein by an anesthetist. Patients were then positioned supine with the right shoulder elevated to 30°. In our center, cardiopulmonary bypass was established by femoral platform. An oblique incision was made usually in the right groin to expose the femoral artery and vein for cannulation. Utilizing the Seldinger technique, the femoral artery was cannulated with a 16 Fr to 20 Fr arterial cannula (Edwards Lifesciences, or Medtronic, USA), and the femoral vein was cannulated with a 24 Fr venous cannula (Edwards Lifesciences). TEE was used to facilitate the placement of the femoral venous cannula up to the superior vena cava.

After initiation of cardiopulmonary bypass, vacuum assistance (10–15 cm H_2_O) was usually used to accomplish total drainage of the atrium. A 5-cm right anterolateral thoracotomy was performed over the fourth intercostal space. After the fourth intercostal space was entered, the lungs were deflated and the right atrium was identified by pushing on the atrial wall with long forceps. Pericardial and atrial tissues were usually bonded tightly as a result of previous operations. Therefore, the pericardium was not dissected from the right atrium, but incised directly. Additionally, an auxiliary operating hole was made around the main incision for continuous carbon dioxide insufflation and passing the traction lines. Using traction lines to expose the right atrium and keeping the balance between arterial inflow and venous drainage, the tricuspid valve was visualized, and replacement or repair was performed on the beating heart with good visual field. After the operation was completed, the original operating hole was used to place the thoracic drainage tube.

### Follow-up

The follow-up was performed either by direct interviews in our outpatient department to evaluate patients’ clinical status or by telephone contact with patients and/or family members. The midterm follow-up was 87.1% complete with a mean duration of 26.3 ± 12.3 months.

### Statistical analysis

Surgical treatment technique was not randomized, but was rather determined by the best medical judgment for each individual case. Data were collected from chart reviews, and were entered into a dedicated Microsoft Excel table.

Categorical variables were represented as frequency distributions and single percentages. Values of continuous variables were expressed as a mean ± standard deviation (SD). Results were considered statistically significant at a p-level below 0.05. All analyses were performed in IBM SPSS statistical package version 26.0 (IBM Corp., Armonk, NY, USA).

## Results

Demographic and clinical characteristics of the patients are depicted in Table 2. Apart from one patient with opening restriction of the artificial mechanical tricuspid valve, all other cases had severe or even tornado tricuspid regurgitation. All patients had clinical manifestations of the right-sided heart failure, including repeated chest tightness and asthma, uncontrolled pedal edema, ascites, and pleural effusion. Their average EuroSCORE was 7.8 ± 1.4 and predicted mortality was nearly 11.2%. Eleven patients belonged to the NYHA functional class IV and 21 patients to the class III. Left ventricular ejection fraction (EF) of patients was almost normal in 58.3 ± 7.1%. In contrast, right ventricular function deteriorated progressively due to severe tricuspid regurgitation, and tricuspid annular plane systolic excursion (TAPSE) was 15.1 ± 2.8 mm. Atrial fibrillation was recorded in 27 patients (84.3%).

**Table 2:** Demographic and clinical characteristics of the patients

Variable	All Patients (*n* = 32)
Age (years)	57.4 ± 8.7 (43 to 72)
Female	18 (56.3%)
NYHA class	
I	0
II	0
III	21 (65.6%)
IV	11 (34.4%)
mean EuroSCORE	7.8 ± 1.4
LVEF	58.3 ± 7.1%
TAPSE (mm)	15.1 ± 2.8
Mean PAP (mmHg)	37.7 ± 11.3
Creatinine level (umol/L)	88.9 ± 31.8
Bilirubin level (umol/L)	24.8 ± 17.3
INR	2.3 ± 0.8
Thrombocytes (10^9)	135.1 ± 55.9
Atrial fibrillation	27 (84.3%)
Atrial flutter	2 (6.3%)
Hypertension	10 (31.3%)
Diabetes mellitus	5 (15.6%)
Preoperative permanent pacemaker	1 (3.1%)

*NYHA* New York Heart Association, *EuroSCORE* European system for cardiac operative risk evaluation, *LVEF* left ventricular ejection fraction, *TAPSE* tricuspid annular plane systolic excusion, *PAP* pulmonary artery pressure, *INR* international standard ratio

Table 3 describes the intraoperative course. All patients underwent minimally invasive beating-heart tricuspid valve replacement or repair (TVR/Rep) without aortic clamping. There were no cases during the operation requiring urgent conversion to the median sternotomy. Thirty patients (93.8%) had their tricuspid valve replaced, whereas two patients (6.2%) had their tricuspid valve repaired. A biological prosthesis was used in all cases when the valve had to be replaced. The average valve size was 30.4 ± 1.2 mm. One patient underwent TVRep with semiflexible ring, and one patient underwent tricuspid valve reconstruction with calf pericardium. Average cardiopulmonary bypass time was 81.5 ± 29.0 min. Intraoperative exploration revealed severe tricuspid regurgitation in 25 patients was caused by enlarged tricuspid annulus and atrioventricular enlargement. A single patient had tricuspid valve limited opening due to artificial mechanical tricuspid valve thrombosis, one had a biological valve failure, while five patients showed organic damage of the valve texture due to previous tricuspid valve surgery. Nine patients (28.1%) needed blood transfusions due to preoperative anemia, and received an average of 0.5 ± 1.3 units of red blood cell (RBC) transfusion.

**Table 3:** Operative characteristics of the patients

Variable	All patients *(n* = 32)
Functional TR	25 (78.1%)
Mechanical valve dysfunction	1 (3.1%)
Biological valve failure	1 (3.1%)
Etiology (previous TVRep)	5 (15.6%)
CPB time (min)	81.5 ± 29.0
TVRep	2 (6.2%)
TVR	30 (93.8%)
Conversion to sternotomy	0
Transfused RBC (units)	0.5 ± 1.3
Transfused Serum (ml)	125.0 ± 236.9

*TR* tricuspid regurgitation, *CPB*, cardiopulmonary bypass, *TVR* tricuspid valve replacement, *TVRep* tricuspid valve repair, *RBC* red blood cell

Postoperative course is described in Table 4. Both in-hospital and overall thirty-day mortalities were 3.1%. Overall in-hospital duration and ICU time were 13.6 ± 7.6 days and 4.1 ± 2.8 days, respectively. Postoperative complications included prolonged ventilation, requiring ventilator-assisted support for more than 72 h in six patients (18.8%). Three patients (9.4%) developed acute renal insufficiency after operation and needed perioperative hemodialysis treatment. One patient (3.1%) suffered permanent neurologic dysfunction, one (3.1%) had a wound infection, and one required postoperative implantation of a permanent pacemaker because of permanent third-degree atrioventricular block. There were no complications such as perioperative myocardial infarction, reoperation for bleeding, mediastinal infection, or other complications. Thirty-one patients were discharged successfully.

**Table 4:** Postoperative outcomes of the patients

Variable	All patients (*n* = 32)
Duration of ICU stay(d)	4.1 ± 2.8
Duration of hospital stay(d)	13.6 ± 7.6
Prolonged ventilation(n)	6 (18.8%)
Reoperation for bleeding	0
Postoperative neurologic event	1 (3.1%)
New-onset renal insufficiency requiring dialysis	3 (9.4%)
Wound infection	1 (3.1%)
In-hospital mortality	1 (3.1%)
30-d mortality	1 (3.1%)
Permanent III atrioventricular block	1 (3.1%)

*ICU* intensive care unit

Table 5 shows the preoperative functional NYHA class of the patients as well as their NYHA status during follow-up after TVR/Rep. The mean midterm follow-up period was 26.3 ± 12.3 months, and the follow-up was complete for 27 patients (87.1%). Four patients were lost to follow-up after TVR or TVRep due to changed contact address. Cardiac function improved significantly in all 27 followed patients. Among them, 23 patients (85.2%) significantly improved to NYHA class I compared with their preoperative status (*p* = 0.000). Three patients improved to NYHA class II compared with their preoperative status (*p* = 0.053). There were no deaths in patients who were followed. One patient underwent radiofrequency ablation for rapid atrial fibrillation. One patient received permanent pacemaker implantation for recurrent syncope due to bradycardia. No reoperations were necessary during the follow-up period.

**Table 5:** Preoperative and postoperative New York Heart Association (NYHA) classification

NYHA Classification	Preoperative (***n*** = 32)	To the present (***n*** = 27)	***p*** value
I	0	23 (85.2%)	0.000
II	0	3 (11.1%)	0.053
III	21 (65.6%)	1 (3.7%)	0.000
IV	11 (34.4%)	0	0.001

## Discussion

We routinely performed redo surgery using a median sternum split before June 2016 with mortality of up to 18.3% [7]. In the present study, we showed that by using simplified, minimally invasive, beating-heart technique, both the mortality and morbidity of redo isolated tricuspid valve surgery were greatly reduced. Most patients were discharged uneventfully. Our favorable early and midterm results are likely related not only to the minimally invasive access, which avoids extensive dissection and minimizes the risk of bleeding, but also to beating-heart technique, which decreases or eliminates ischemia reperfusion injury that follows standard maneuvers of aortic cross-clamping and clamp release. However, for patients undergoing reoperative surgery, the traditional median sternotomy approach is associated with a high risk of bleeding and risk of heart injury. In addition, obese and diabetic patients are particularly prone to sternal infection and sternal instability [13]. Although minimally invasive technique has been reported elsewhere [14–17], the procedure as conducted in our center includes many specific technical details. It is reasonable to expect that every technical detail contributes to the success of an operation. In our center, all patients were routinely implanted with endocardial temporary pacing lead through right internal jugular vein by the anesthetist before the operation, because the walking path of the conduction beam is very close to the operation area and nobody can surely avoid the third-degree atrioventricular block for tricuspid valve replacement. To provide a bloodless working environment, some surgeons prefer using bicaval cannulation techniques with caval tapes or balloon [10, 18, 19]. We chose the “one incision, two cannulas” technique [20] for all of our cases. Femoral venous cannula was inserted up to the superior vena cava under the assistance of TEE. The positions of the superior and inferior vena cava were at a lower point compared with the tricuspid valve position. Therefore, there was no need to worry that a large amount of gas would enter the venous drainage line [21]. Meanwhile, a vacuum was also used to achieve a good venous drainage. By applying these techniques, we easily achieved a bloodless surgical field.

Some early reports showed a lower incidence of stroke with aortic versus femoral artery cannulation. The reason may be that peripheral cannulation increases the incidence of retrograde embolism from the descending thoracoabdominal aorta. However, femoral artery cannulation was routinely established for minimally invasive surgeries in our center, and stroke occurred in only one patient in the present series. Likewise, Lamelas evaluated all minimally invasive cardiac procedures that utilized femoral arterial cannulation over the past 6 years, and reported acceptably low incidences of stroke (1.17%) and peripheral arterial trauma (0.07%; e.g., dissection, pseudoaneurysm, and fistula) [22]. Nifong et al. also reported low stroke risk with femoral artery cannulation [23]. Shann and Melnitchouk reported that the femoral artery cannulation is often used for procedures that utilize a right anterolateral minithoracotomy incision [24]. Overall, the main principle of minimally invasive surgery should leave the limited view for surgical procedures to the most degree by the establishment of peripheral CPB.

In our series, although most of redo tricuspid regurgitation was due to functional change, 93.8% (30/32) of patients underwent tricuspid valve replacement instead of repair. Considering characteristics of our patients, such as younger mean age, previous cardiac surgery, chronic atrial fibrillation, and severe congestive heart failure (NYHA III–IV), we think that tricuspid valve replacement is the best option to avoid recurrent tricuspid regurgitation. We agree with Pfannmuller’s opinion, and we also routinely use biological prostheses, regardless of a patient’s age, even in patients with a previously implanted mechanical valve (regardless of whether it is in the aortic or mitral position, or both) to avoid trouble due to endocardial temporary pacing lead implantation and excessive anticoagulation mandatory for a mechanical prosthesis in a tricuspid position [25]. While some researchers reported that tricuspid valve replacement was always associated with significantly higher mortality [1, 16], others found no statistically significant differences in early and late outcomes between the isolated tricuspid valve repair and replacement surgeries [6]. However, one study reported a reduced mortality with the replacement surgery, which is in accordance with our results [3]. The choice of replacement or repair should be based on the surgeons’ comprehensive judgment of the patient’s overall state as well as personal experience.

### Study limitations

There are several limitations of this study. First, it was only a retrospective, clinical observational trial in a single center, which has likely led to bias in patient selection and technique used. For final confirmation of our results, a prospective, multi-center study involving a larger sample size is required. Secondly, the small size of our series (only 32 patients) may influence generalizability of our findings. Additionally, although the early and midterm follow-up results are excellent, we do not yet have long-term follow-up data with our present technique. Finally, surgical treatment technique was not randomized, but rather was determined by the best medical judgment for each individual case. Therefore, we do not have an appropriate control group.

## Conclusions

In this series of high-risk patients, redo tricuspid valve operation with a simplified, minimally invasive, beating-heart technique was both feasible and safe. The approach was associated with encouraging early and midterm results, showing low mortality and morbidity. Further studies are required to allow a direct comparison between our present technique and median sternotomy. Long-term data are also needed regarding the durability of these procedures.

## Data Availability

Not applicable.

## Abbreviations

CPB: Cardiopulmonary bypass
EuroSCORE: European system for cardiac operative risk evaluation
ICU: Intensive care unit
INR: International standard ratio
LVEF: Left ventricular ejection fraction
NYHA: New York Heart Association
PAP: Pulmonary artery pressure
RBC: Red blood cell
TAPSE: Tricuspid annular plane systolic excursion;
TR: Tricuspid regurgitation
TVR: Tricuspid valve replacement
TVRep: Tricuspid valve repair
